# Exploring the brains of *Baduk* (*Go*) experts: gray matter morphometry, resting-state functional connectivity, and graph theoretical analysis

**DOI:** 10.3389/fnhum.2013.00633

**Published:** 2013-10-02

**Authors:** Wi Hoon Jung, Sung Nyun Kim, Tae Young Lee, Joon Hwan Jang, Chi-Hoon Choi, Do-Hyung Kang, Jun Soo Kwon

**Affiliations:** ^1^Department of Psychiatry, Clinical Cognitive Neuroscience Center, SNU-MRCSeoul, South Korea; ^2^Department of Psychiatry, Seoul National University College of MedicineSeoul, South Korea; ^3^Department of Diagnostic Radiology, National Medical CenterSeoul, South Korea; ^4^Brain and Cognitive Sciences-WCU Program, College of Natural Sciences, Seoul National UniversitySeoul, South Korea

**Keywords:** amygdala, *Baduk*, head of caudate, intuitive judgment, resting-state functional connectivity, somatic marker hypothesis, voxel-based morphometry

## Abstract

One major characteristic of experts is intuitive judgment, which is an automatic process whereby patterns stored in memory through long-term training are recognized. Indeed, long-term training may influence brain structure and function. A recent study revealed that chess experts at rest showed differences in structure and functional connectivity (FC) in the head of caudate, which is associated with rapid best next-move generation. However, less is known about the structure and function of the brains of *Baduk* experts (BEs) compared with those of experts in other strategy games. Therefore, we performed voxel-based morphometry (VBM) and FC analyses in BEs to investigate structural brain differences and to clarify the influence of these differences on functional interactions. We also conducted graph theoretical analysis (GTA) to explore the topological organization of whole-brain functional networks. Compared to novices, BEs exhibited decreased and increased gray matter volume (GMV) in the amygdala and nucleus accumbens (NA), respectively. We also found increased FC between the amygdala and medial orbitofrontal cortex (mOFC) and decreased FC between the NA and medial prefrontal cortex (mPFC). Further GTA revealed differences in measures of the integration of the network and in the regional nodal characteristics of various brain regions activated during *Baduk*. This study provides evidence for structural and functional differences as well as altered topological organization of the whole-brain functional networks in BEs. Our findings also offer novel suggestions about the cognitive mechanisms behind *Baduk* expertise, which involves intuitive decision-making mediated by somatic marker circuitry and visuospatial processing.

## Introduction

Board games such as chess have been studied by researchers from a variety of fields, such as economics (Levitt et al., 2011), computer science (Bouzy and Cazenave, 2001; Cai et al., 2010), and cognitive science (de Groot, 1965; Chase and Simon, 1973), because of the similarity between board games and real life in terms of the need to engage in decision-making and adaptive behavior to achieve specific goals under changing environmental conditions. Cognitive science, in particular, has used board games to study cognitive expertise, as playing involves diverse cognitive functions such as attention, working memory, visuospatial processing, and decision-making (Chase and Simon, 1973; Gobet and Charness, 2006). Board-game players with the highest level of skill, known as grand masters, are considered cognitive experts who develop the knowledge structures used in problem solving in a given domain through long periods of deliberate practice (Chase and Simon, 1973). Using these knowledge structures, called chunks, templates, or schemas (Chase and Simon, 1973; Gobet and Charness, 2006), experts can rapidly match the patterns they have learned and make faster and better decisions. Such chunk-driven unconscious automatic cognitive processes are often referred to as intuition, which is defined as the recognition of patterns or structures stored in long-term memory (Chase and Simon, 1973), and a number of researchers have proposed accounts of the mechanisms underlying intuitive judgment (Hodgkinson et al., 2009; Minavand chal et al., 2013), such as the following: dual-process theory, naturalistic decision-making (NDM), and somatic marker hypothesis (SMH). For example, the recognition-primed decision (RPD) model within the NDM approach focuses on the success of expert intuition (de Groot, 1965; Klein, 1998, 2008), as opposed to the heuristic-and-biases approach which adopts a skeptical attitude toward expert judgment (Kahneman and Klein, 2009). This shows how experts can make extremely rapid and favorable decisions by combining two processes: (i) an intuitive (automatic) process involving pattern matching based on past experience and (ii) a deliberative (conscious) process involving mental simulation (or analysis) to imagine how a course of action will play out (Klein, 1993; Kahneman and Klein, 2009). The SMH emphasizes the influence of emotion-based signals (somatic states) emerging from the body, such as gut feelings on intuitive decision-making (Damasio, 1996; Dunn et al., 2006). Despite previous extensive studies on the mechanism behind intuitive expertise in board games, its neural basis remained largely enigmatic until the last two decades (Nichelli et al., 1994). Recent brain imaging studies during board-game play have resulted in renewed interest in the neural basis of cognitive expertise and have revealed brain regions associated with object recognition, such as the lateral occipital complex, occipitotemporal junction, (Bilalić et al., 2011a,b) and the fusiform cortex (FFC) (Bilalić et al., 2011c), with pattern recognition, such as the collateral sulcus (CoS) and retrosplenial cortex (RSC) (Bilalić et al., 2010, 2011b), with recognition of relations between objects, such as the supramarginal gyrus (SMG) (Bilalić et al., 2011a,b), and with intuitive best next-move generation during chess play, such as the head of the caudate (HOC) (Wan et al., 2011, 2012). However, most neuroimaging studies with board-game experts have involved chess, even though *Baduk* differs fundamentally from chess in terms of the mental strategies involved.

*Baduk*, as it is known in Korean (*Go* in Japanese and *Weiqi* in Chinese), is a popular board game in East Asia; it is played on a square board consisting of a pattern of 19 by 19 crossed lines. Whereas chess pieces have specific identities and functions, all *Baduk* pieces (called stones) have the same value and function. Rules of the game are very simple (http://english.Baduk.or.kr); two players, one playing with black stones and the other playing with white ones, alternately place a stone to capture as large an area as possible on the board by surrounding the opponent's stones. Despite its simple rules, *Baduk* is characterized by greater combinatorial complexity than chess due to the tremendous size of its game tree; the average branching factor (i.e., the number of move choices available per turn) is approximately 200 in *Baduk*, whereas it is about 35 in chess (Keene and Levy, 1991). Additionally, unlike most other strategy games, *Baduk* cannot be won by a computer program, whereas computerized chess programs can beat even the world's best human player (Bouzy and Cazenave, 2001). Although chess and *Baduk* share common cognitive and affective processes, such as memory, attention, perception, and emotional regulation, the two games nonetheless differ in the following important ways. Given its larger game tree and heavy dependence on spatial positioning rather than on selecting pieces according to their roles, knowledge and pattern recognition with respect to spatial positioning may be more important in *Baduk* than in other strategy games (Gobet et al., 2004). Recent neuroimaging studies on *Baduk* experts (BEs) have demonstrated increased activity in the occipitotemporal and parietal cortices, areas associated with visuospatial processing, such as integration of local features (Kourtzi et al., 2003) and spatial attention (Fink et al., 1996) respectively, while performing *Baduk* tasks (Chen et al., 2003; Ouchi et al., 2005). In addition to cognitive competences such as spatial processing, researchers have recently emphasized emotional processing in competitive board-game (Grabner et al., 2007) because based on evidence for the SMH (Bechara et al., 1994; Blakemore and Robbins, 2012), our performance (i.e., decision-making) is strongly affected by emotions. Thus, since board-game players experience a variety of emotions while playing, an imbalance in the emotions can cause mistakes (DeGroot and Broekens, 2003). Accumulated evidence from neuroimaging and lesion studies implicates the amygdala (AMY), striatum, and orbitofrontal cortex in emotional processing (Phillips et al., 2003a,b), and suggests the ventromedial prefrontal cortex (vmPFC), AMY, somatosensory cortex, and insula as regions of brain circuitry involved in the SMH (Damasio, 1996; Dunn et al., 2006). Particularly, the vmPFC is thought to play a role in generating somatic markers (Damasio, 1996). Taken together, BEs may show differences in morphology and/or function in brain regions associated with spatial processing and emotion-based decision-making. However, until recently, there have not been studies investigating whether such specific differences exist in the brains of long-term trained BEs.

Many neuroimaging studies about the learning- and practice-based superior performance of experts have provided evidence for cross-sectional differences and longitudinal changes in brain structure and function, known as neuroplasticity, in brain areas underlying specific skills. Such brain areas include the occipito–temporal cortex, which is associated with complex visual motions in jugglers (Draganski et al., 2004), the hippocampus, which is associated with spatial learning and memory in taxi drivers (Maguire et al., 2000; Spiers and Maguire, 2006), and the medial prefrontal cortex (mPFC)/medial orbitofrontal cortex (mOFC), which is associated with emotion regulation and self-referential processing in meditation experts (Jang et al., 2011; Kang et al., 2013). In particular, recent studies have revealed that, compared to novices, chess experts demonstrate morphological differences in the HOC and its influence on functional circuits, showing a decrease in gray matter volume (GMV) and an increase in functional connectivity (FC) in this region during resting-state (Duan et al., 2012). However, whether such brain differences are specific to chess experts or extend to experts in other strategy games remains unclear. To address this issue, we used voxel-based morphometry (VBM) and resting-state functional connectivity (RSFC) analysis to compare BEs and novices in terms of GMV and to examine the effects of these morphological differences on functional brain connectivity at rest.

RSFC analysis based on resting-state functional magnetic resonance imaging (rs-fMRI) reveals spontaneous or intrinsic functional connections of the brain, which are reflected in the correlation pattern of low-frequency blood-oxygen-level-dependent (BOLD) fluctuations between small regions of interest and all other brain regions (Fox et al., 2005). Recently, this approach has been extensively used in conjunction with graph theory to investigate the topological organization of brain networks (Wang et al., 2010). Graph theoretical analysis (GTA) of rs-fMRI enables visualization of the overall connectivity pattern across all brain regions and provides quantitative measurement of complex patterns of organization across a network, such as small-worldness, which measures global network connection efficiency. Using this approach, recent studies have reported differences in topological organization of the whole-brain functional network between personality dimensions of extraversion and neuroticism (Gao et al., 2013), as well as between various brain diseases that involve cognitive impairments, such as Alzheimer's disease (Supekar et al., 2008) and schizophrenia (Lynall et al., 2010), and healthy controls. However, the topological organization of the whole-brain functional network in cognitive experts is yet to be elucidated.

We hypothesized that BEs would exhibit morphological differences in brain regions underlying expertise in *Baduk*, particularly the occipitotemporal and parietal areas associated with visuospatial processing and spatial attention respectively, as well as the somatic marker circuitry involved in emotion-based decision-making, and that these morphological differences may be associated with alterations in the functional circuits of these regions. We also predicted that the topological organization of their whole-brain functional network would be altered in the service of achieving the most efficient network for playing *Baduk*. To test our hypotheses, we employed VBM and RSFC, and further analyzed the topological properties of the intrinsic brain connectivity network using a graph theoretical approach. We expect that this study will provide evidence for structural and functional brain differences in BEs, as well as offer additional insight into the nature of the varied and complex cognitive mechanisms that enable superior performance by BEs.

## Materials and methods

### Participants

Seventeen BEs who had been training for 12.47 ± 1.50 years were recruited from the Korea *Baduk* Association (http://english.Baduk.or.kr/). BEs experts were statistically matched for age, sex, and education level to 16 novices who knew the rules for playing *Baduk*. All subjects were right handed and had no history of neurological or psychiatric problems. The demographic characteristics of each group are presented in Table 1. All procedures performed in this study were approved by the Institutional Review Board of Seoul National University Hospital.

**Table 1:** **Demographic characteristics of experts and novices**.

	**Experts (*n* = 17)**	**Novices (*n* = 16)**	***p-*value**
Sex (male/female)^†^	14/3	12/4	0.606
Age (years)^‡^	17.177 (1.131)	16.938 (1.124)	0.547
Education (years)^‡^	9.647 (2.805)	10.688 (1.302)	0.186
IQ^‡^	93.118 (10.093)	100.750 (12.503)	0.062
Training duration (years)	12.471 (1.505)	–	–

IQ, intelligence quotient. Values are presented as mean (standard deviation).

† *χ^2^ test was used*.

‡ Independent t-test was used.

### Data acquisition

All image data were acquired using a 1.5-T scanner (Siemens Avanto, Germany). High-resolution anatomical images of the whole brain were acquired with T1-weighted 3-D magnetization-prepared rapid-acquisition gradient-echo (MPRAGE) sequence [repetition time (TR)/echo time (TE) = 1160/4.76 ms, flip angle = 15°, field of view (FOV) = 230 mm, matrix size = 256 × 256]. rs-fMRI data were obtained via a gradient echo-planar imaging pulse sequence (TR/TE = 2340/52 ms, flip angle = 90°, FOV = 220 mm, voxel size = 3.44 × 3.44 × 5 mm^3^), during which subjects were instructed to relax with their eyes closed without falling asleep. rs-fMRI scans were part of fMRI sessions, during which participants performed working memory tasks. Resting-state runs were performed for 4.68-min (120 volumes) prior to administration of the working memory tasks. Other image parameters (task-related fMRI and DTI) that are not related to the present study are not described herein. Based on visual inspection, a neuroradiologist (CHC) judged all scans to be excellent, without obvious motion artifacts, signal loss, or gross pathology.

### Voxel-based morphometry analysis

T1 data were processed using VBM8 toolbox (http://dbm.neuro.uni-jena.de/vbm.html) implemented in SPM8 (http://www.fil.ion.ucl.ac.uk/spm), with default parameters incorporating the DARTEL toolbox to produce a high-dimensional normalization protocol (Ashburner, 2007). Images were corrected for bias-field inhomogeneities, tissue-classified into gray matter (GM), white matter (WM), and cerebrospinal fluid (CSF) based on unified segmentation from SPM8, and spatially normalized to the MNI space using linear (12-parameter affine) and non-linear transformations (warping). The nonlinear transformation parameters were calculated via the DARTEL algorithm (Ashburner, 2007) with an existing standard template in VBM8. The warped GM segments were modified to compensate for volume changes during spatial normalization by multiplying the intensity value in each voxel by the Jacobian determinants (modulated GMVs). Finally, the resulting GM images were smoothed with an 8-mm full-width at half maximum (FWHM) isotropic Gaussian kernel. Voxel-wise comparisons of GMV in the two groups were performed using two-sample *t*-tests. Total intracranial volume (TIV) was modeled as a covariate of no interest. TIV was calculated by summing the raw volumes of GM, WM, and CSF, in which each tissue volume was automatically generated as a text file for each subject (^*^_seg8.txt) in VBM8 processing. The statistical significance of group differences was set at *p* < 0.05 using AlphaSim correction (with a combination of a threshold of *p* < 0.005 and a minimum cluster size of 340 voxels) (Cox, 1996). Based on previous research (Duan et al., 2012), a looser p-threshold was chosen (*p* < 0.005 and expected voxels per cluster *k* > 133) to detect the presence of group differences in the HOC. To investigate associations between the GMV of BEs and training duration, we employed SPM8 to perform voxel-wise correlation analysis between these two values using a multiple regression model.

### Functional connectivity analysis

**Figure 1:**
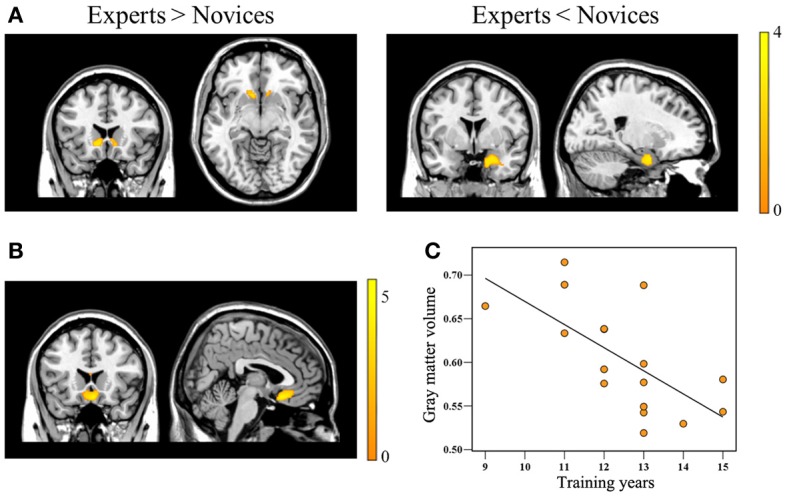
**Brain regions showing significant group differences in gray matter volume and showing a correlation with *Baduk* training years.** Relative to novices, *Baduk* experts showed significantly increased gray matter volume in the bilateral caudate, particularly the nucleus accumbens, and significantly decreased gray matter volume in the right amygdala (panel **A**). Experts showed significantly negative correlations between gray matter volume in the medial orbitofrontal cortex (mOFC) adjacent to the gyrus rectus and their training years (panels **B,C**).

**Figure 2:**
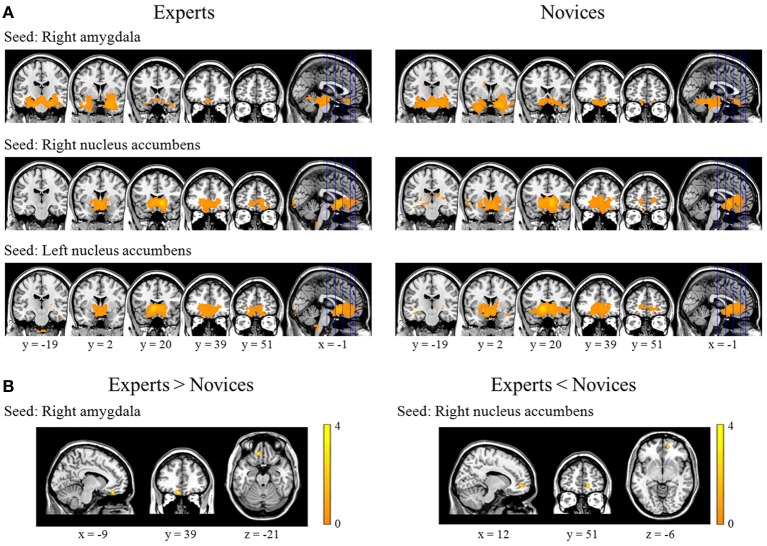
**Results from within- and between-group analyses of resting-state functional connectivity.** These figures provide significantly positive correlation maps for the right amygdala, right nucleus accumbens, and left nucleus accumbens as seed regions in experts (the left column) and novices (the right column) (panel **A**). Experts displayed significantly increased functional connectivity between the right amygdala and left medial orbitofrontal cortex, and significantly decreased connectivity between the right nucleus accumbens and right medial prefrontal cortex (panel **B**).

rs-fMRI data preprocessing was performed using SPM8 and REST V1.7 toolkit (http://www.restfmri.net/; Song et al., 2011). The preprocessing procedures for rs-fMRI data were performed as follows. After discarding the first four volumes to allow for stabilization of the BOLD signal, each subject's rs-fMRI data were (i) corrected for slice-timing differences, (ii) realigned to their first scan to correct for movement, (iii) spatially normalized to the MNI echo-planar imaging template in SPM8 (voxels were resampled to 3 × 3 × 3 mm^3^), (iv) spatially smoothed with a 6-mm FWHM Gaussian kernel, (v) removed of the linear trend of time courses, (vi) temporally band-pass filtered (0.01–0.08 Hz), and (vii) conducted regression of nuisance signals (head-motion profiles, global signal, WM, and CSF) to correct for physiological noises. Regions showing significant group differences in GMV according to the VBM results were defined as seed regions for subsequent FC analysis [i.e., right AMY, right and left nucleus accumbens (NA); Figure 1A]. FC maps were produced by extracting the time series averaged across voxels within each seed region and then computing the Pearson's correlation between that time series and those from all other brain voxels. Finally, correlation coefficients for each voxel were converted into a normal distribution by Fischer's *z* transform (Fox et al., 2005). For each group, individual *z*-value maps were analyzed with a random-effect one-sample *t*-test to identify voxels with a significant positive correlation to the seed time series, which correlations threshold at *p* < 0.001, uncorrected, and a topological false-discovery-rate (FDR) correction threshold at *p* < 0.05 for multiple comparisons (Figure 2A; Chumbley et al., 2010). For between-group comparisons, two-sample *t*-tests were used to compare *z*-value maps between experts and novices using AlphaSim correction with significance set at *p* < 0.05 (with a combination of a threshold of *p* < 0.005 and a minimum cluster size of 13 voxels for each mask map) (Table 2). This analysis was restricted to the voxels showing significant positive correlation maps for either experts or novices by using an explicit mask from the combined sets of the results of the one-sample *t*-tests (*p* < 0.05, topological FDR corrected) of the two groups.

**Table 2:** **Regions showing significant group differences in gray matter volume and functional connectivity**.

**Regions**	**MNI coordinates**	**Experts (*n* = 17)^†^**	**Novices (*n* = 16)^†^**	***t*-value**	***z*-value**
**VOXEL-BASED MORPHOMETRY RESULTS**					
*Experts > Novices*					
Right nucleus accumbens	9, 21, −6	0.428 (0.027)	0.382 (0.038)	3.47	3.16
Left nucleus accumbens	−11, 20, −8	0.503 (0.030)	0.453 (0.041)	3.45	3.14
*Experts < Novices*					
Right amygdala	21, 2, −24	0.541 (0.055)	0.573 (0.031)	−3.53	3.20
*Negative correlation between gray matter volumes and training duration*					
Right medial orbitofrontal cortex^‡^	3, 20, −17	0.604 (0.061)	–	5.26	3.84
**RESTING-STATE FUNCTIONAL CONNECTIVITY RESULTS**					
*Experts > Novices*					
Right amygdala seed					
Left medial orbitofrontal cortex	−9, 39, −21	0.276 (0.136)	0.078 (0.137)	3.68	3.32
*Experts < Novices*					
Right nucleus accumbens seed					
Right medial prefrontal cortex	12, 51, −6	0.102 (0.120)	0.321 (0.178)	−3.95	3.53

† Values are presented as mean beta values (standard deviation) for each group; mean beta values for each subject were extracted from each region using MarsBaR toolbox for SPM (http://marsbar.sourceforge.net/)

‡ We performed a correlation analysis between beta value extracted from the region of and training duration of each subject using SPSS (p < 0.001, r = −0.802).

### Network construction and analysis

**Figure 3:**
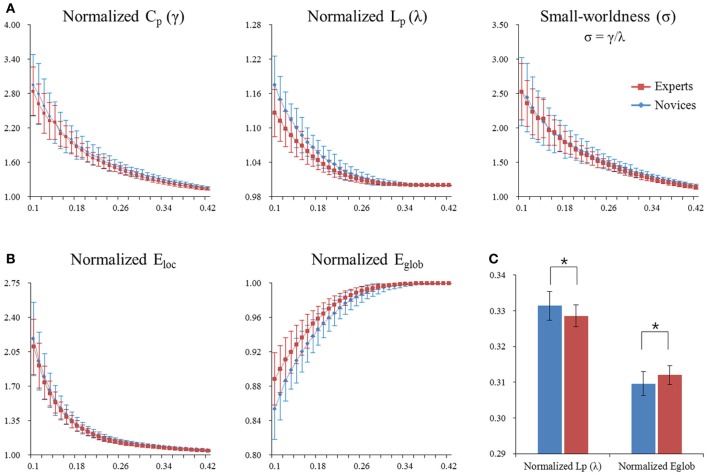
**Normalized global network measures of the whole-brain functional network in both groups.** The global measures included small-word parameters (panel **A**) and network efficiency (panel **B**). Asterisks denote significant differences (permutation-based *p*-value < 0.05). Significant differences were found in normalized path length λ (permutation-based *p*-value = 0.018) and normalized global efficiency *E*_glob_ (permutation-based *p*-value = 0.008) between experts and novices (panel **C**). Error bars denote standard deviations. *C*_*P*_, clustering coefficient; *L*_*P*_, characteristic path length; γ, normalized clustering coefficient; λ, normalized characteristic path length; σ, small-worldness; *E*_loc_, local efficiency; *E*_glob_, global efficient.

**Figure 4:**
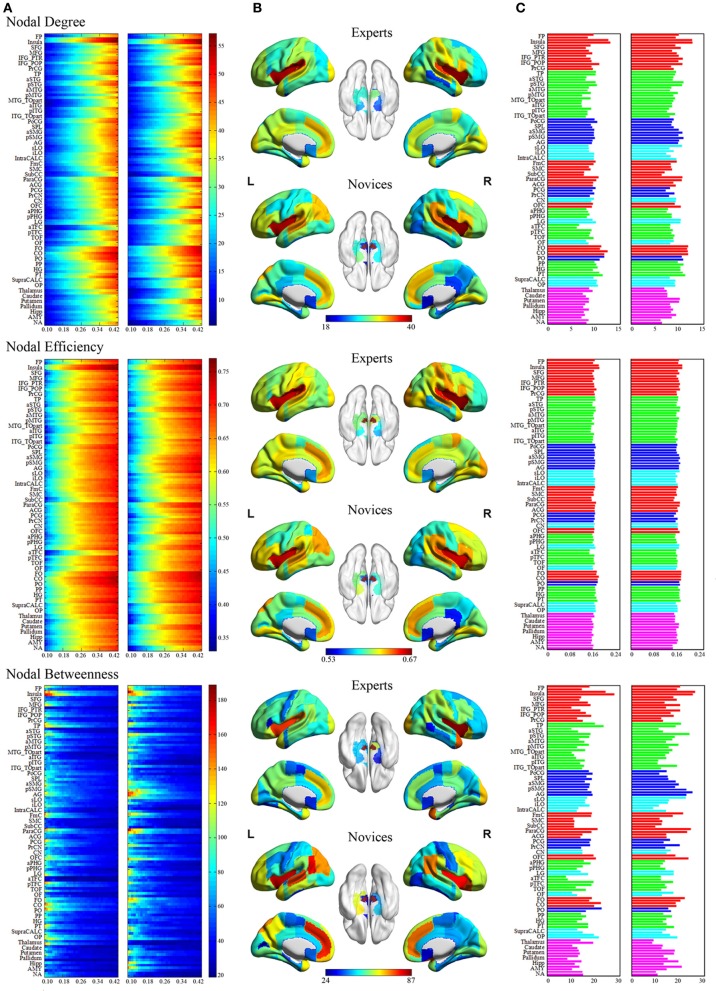
**Regional network measures (i.e., nodal degree, nodal efficiency, and nodal betweenness) for experts and novices.** (Panel **A**) shows values of each nodal metric over a range of thresholds in each group. (Panel **B**) shows mean values for each nodal metric across a range of thresholds in each group, which were superimposed on an inflated standard brain using BrainNet Viewer (http://www.nitrc.org/projects/bnv/). (Panel **C**) shows a bar plot of the AUC values of each nodal metric (red, frontal areas; green, temporal areas; blue, parietal areas; sky, occipital areas; purple, subcortical areas).

In this study, brain networks were composed of nodes representing brain regions and edges representing interregional RSFC. To define network nodes, the Harvard–Oxford atlas (HOA) was employed to divide the whole brain, excluding the brainstem, into 110 (55 for each hemisphere) cortical and subcortical regions-of-interest (ROIs) (Table 3). To define the network edges, we calculated the Pearson correlations between pairs of ROIs. Correlation matrices were thresholded into binary networks, applying network sparsity (*S*) (the ratio of the number of existing edges divided by the maximum number of possible edges in a network). The sparsity threshold was normalized so that each group network had the same number of nodes and edges, allowing investigation of the relative network efficiency of each group (Achard and Bullmore, 2007). Given the absence of a gold standard for selecting a single threshold, based on previous studies (Wang et al., 2010; Tian et al., 2011), a continuous range of 0.10 ≤ *S* ≤ 0.42 with an interval of 0.01 was employed to threshold the correlation matrices into a set of binary matrices. This range of sparsity allows prominent small-world properties of brain networks to be observed (Watts and Strogatz, 1998); that is, the small-worldness of the thresholded networks was larger than 1.1 for all participants (Zhang et al., 2011; Gao et al., 2013). We calculated both global and regional network measures of brain networks at each sparsity threshold (Figures 3A,B, 4A,B). The global measures included (i) small-world parameters (clustering coefficient *C*_*P*_, characteristic path length *L*_*P*_, and small-worldness σ) and (ii) network efficiency (local efficiency *E*_loc_ and global efficiency *E*_glob_). The regional measures included three nodal centrality metrics: degree, efficiency, and betweenness (Rubinov and Sporns, 2010; Tian et al., 2011). In this study, we calculated all these metrics using GRETNA v1.0 (https://www.nitrc.org/projects/gretna/), which is a graph-theoretical network analysis toolkit, with PSOM (Pipeline System for Octave and Matlab, (http://code.google.com/p/psom) and MatlabBGL package (http://www.cs.purdue.edu/homes/dgleich/packages/matlab_bgl/). Mathematical explanations for each network metric are provided in the following sub-sections.

**Table 3:** **Anatomic regions-of-interest included in the network analysis^†^**.

**Regions**	**Abbreviation**	**Classification^‡^**	**ROI index**	
			**Left hemisphere**	**Right hemisphere**
Frontal pole	FP	frontal	1	2
Insular cortex	Insula	frontal	3	4
Superior frontal gyrus	SFG	frontal	5	6
Middle frontal gyrus	MFG	frontal	7	8
Inferior frontal gyrus, pars triangularis	IFG_PTR	frontal	9	10
Inferior frontal gyrus, pars opercularis	IFG_POP	frontal	11	12
Precentral gyrus	PrCG	frontal	13	14
Temporal pole	TP	temporal	15	16
Superior temporal gyrus, anterior division	aSTG	temporal	17	18
Superior temporal gyrus, posterior division	pSTG	temporal	19	20
Middle temporal gyrus, anterior division	aMTG	temporal	21	22
Middle temporal gyrus, posterior division	pMTG	temporal	23	24
Middle temporal gyrus, temporooccipital part	MTG_TOpart	temporal	25	26
Inferior temporal gyrus, anterior division	aITG	temporal	27	28
Inferior temporal gyrus, posterior division	pITG	temporal	29	30
Inferior temporal gyrus, temporooccipital part	ITG_TOpart	temporal	31	32
Postcentral gyrus	PoCG	parietal	33	34
Superior parietal lobule	SPL	parietal	35	36
Supramarginal gyrus, anterior division	aSMG	parietal	37	38
Supramarginal gyrus, posterior division	pSMG	parietal	39	40
Angular gyrus	AG	parietal	41	42
Lateral occipital cortex, superior division	sLO	occipital	43	44
Lateral occipital cortex, inferior division	iLO	occipital	45	46
Intracalcarine cortex	IntraCALC	occipital	47	48
Frontal medial cortex	FmC	frontal	49	50
Supplementary motor cortex	SMC	frontal	51	52
Subcallosal cortex	SubCC	frontal	53	54
Paracingulate gyrus	ParaCG	frontal	55	56
Cingulate gyrus, anterior division	ACG	frontal	57	58
Cingulate gyrus, posterior division	PCG	parietal	59	60
Precuneous cortex	PrCN	parietal	61	62
Cuneal cortex	CN	occipital	63	64
Frontal orbital cortex	OFC	frontal	65	66
Parahippocampal gyrus, anterior division	aPHG	temporal	67	68
Parahippocampal gyrus, posterior division	pPHG	temporal	69	70
Lingual gyrus	LG	occipital	71	72
Temporal fusiform cortex, anterior division	aTFC	temporal	73	74
Temporal fusiform cortex, posterior division	pTFC	temporal	75	76
Temporal occipital fusiform cortex	TOF	temporal	77	78
Occipital fusiform gyrus	OF	occipital	79	80
Frontal operculum cortex	FO	frontal	81	82
Central opercular cortex	CO	frontal	83	84
Parietal operculum cortex	PO	parietal	85	86
Planum polare	PP	temporal	87	88
Heschl's gyrus	HG	temporal	89	90
Planum temporale	PT	temporal	91	92
Supracalcarine cortex	SupraCALC	occipital	93	94
Occipital pole	OP	occipital	95	96
Thalamus	Thalamus	subcortical	97	98
Caudate	Caudate	subcortical	99	100
Putamen	Putamen	subcortical	101	102
Pallidum	Pallidum	subcortical	103	104
Hippocampus	Hipp	subcortical	105	106
Amygdala	AMY	subcortical	107	108
Nucleus accumbens	NA	subcortical	109	110

† *To define network nodes, the Harvard-Oxford atlas (HOA) was employed to divide the whole brain into 110 (55 for each hemisphere) cortical and subcortical regions of interest (ROIs), except the brainstem*.

‡ *To facilitate data characterization and interpretation, we sorted nodes based on lobar (i.e., frontal, temporal, parietal, occipital, and subcortical) classification*.

#### Global network parameters

To characterize the global topological organization of whole-brain functional network, we considered five network metrics: clustering coefficient (*C*_*P*_), characteristic path length (*L*_*P*_), small-worldness (σ), global efficiency (*E*_glob_), and local efficiency (*E*_loc_). *C*_*P*_ indicates how well neighbors of a node *i* are connected (i.e., local interconnectivity of a network). *L*_*P*_ is the shortest path length (i.e., number of edges) required to transfer from one node to another averaged over all pairs of nodes. *E*_glob_ is a measure of the capacity for parallel information transfer over the network, and is inversely related to *L*_*P*_. *E*_loc_ is a measure of the fault tolerance of the network, indicating how well each subgraph exchanges information when the index node is eliminated, and is related to *C*_*P*_. While high *E*_loc_ and *C*_*P*_ reflect a high local specialization (called segregation) of information processing, high *E*_glob_ and low *L*_*P*_ express a great ability to integrate information from the network.

For a given graph *G* with *N* nodes, the clustering coefficient is defined by Watts and Strogatz (1998) as:

$$C_{P}(G)=\frac{1}{N}\sum_{i\in G}\frac{E_{i}}{D_{\text{nod}}(i)(D_{\text{nod}}(i)-1)/2},$$

where *D*_nod_(i) (see below) is the degree of a node *i*, and *E*_*i*_ is the number of edges in *G*_*i*_, the subgraph consisting of the neighbors of a node *i*. The characteristic path length is defined by Newman (2003) as:

$$L_{P}(G)=\frac{1}{\frac{1}{N(N-1)}\left(\sum_{j\neq \in G}{\frac{1}{L}}_{ij}\right)},$$

where *L*_*ij*_ is the shortest path length between nodes *i* and *j*. To examine the small-world properties, the values of *C*_*P*_ and *L*_*P*_ were normalized as compared with those of 100 degree-matched random networks (γ = *C*^real^_*P*_/*C*^rand^_*P*_and λ = *L*^real^_*P*_/*L*^rand^_*P*_, σ = γ/λ) before statistical analysis (Maslov and Sneppen, 2002). Typically, a small-world network should meet the following conditions: γ > 1 and λ ≈ 1 (Watts and Strogatz, 1998), or σ = γ/λ > 1 (Humphries et al., 2006). The global efficiency of *G* is defined by Latora and Marchiori (2001) as:

$$E_{\text{glob}}(G)=\frac{1}{N(N-1)}\sum_{j\neq i\in G}{\frac{1}{L}}_{ij},$$

The local efficiency of G is defined by Latora and Marchiori (2001) as:

$$E_{\text{loc}}(G)=\frac{1}{N}\sum_{i\in G}E_{\text{glob}}(G_{i}),$$

where *E*_glob_(*G*_*i*_)is the global efficiency of *G*_*i*_, the subgraph composed of the neighbors of a node *i*.

#### Regional nodal parameters

To investigate the regional characteristics of whole-brain functional network, we considered three nodal metrics: the nodal degree (*D*_nod_), the nodal efficiency (*E*_nod_), and the nodal betweenness (*B*_nod_). All these nodal metrics detect the importance of individual nodes in the network. *D*_nod_ measures the connectivity of a node *i* with all other nodes in the whole brain. That is, nodes with high degree interact with many other nodes in the network. *E*_nod_ measures the information propagation ability of a node *i* with the all other nodes in the whole brain. *B*_nod_ measures the influence of a node *i* over information flow between all other nodes in the whole network. That is, it is the fraction of all shortest paths in the network that pass through a given node. The nodal degree of a node *i* is defined as:

$$D_{\text{nod}}(i)=\sum_{j\neq i\in G}e_{ij},$$

where *e*_*ij*_ is the *i* th and *j* th column element of the obtained binarized correlation matrix. The normal efficiency of a node *i* is defined as Achard and Bullmore (2007):

$$E_{\text{nod}}(i)=\frac{1}{N-1}\sum_{j\neq i\in G}{\frac{1}{L}}_{ij},$$

The betweenness of a node *i* is defined as Freeman (1977):

$$B_{\text{nod}}(i)=\sum_{j\neq i\neq k\in G}\frac{\delta_{jk}(i)}{\delta_{jk}},$$

where δ_*jk*_ is the number of shortest paths from a node *j* to a node *k*, and δ_*jk*_(*i*) is the number of shortest paths from a node *j* to a node *k* that pass through a node *i* within graph *G*.

#### Statistical analysis in network parameters

For statistical comparisons of the two groups, we calculated the area under the curve (AUC) for each network metric, which yields a summarized scalar to integrate the topological characteristics of brain networks over a range of thresholds (Figures 3C, 4C). Between-group differences in each measure were inferred by nonparametric permutation tests (5000 permutations) for the AUC of each global and regional measure. Based on a previous study (Zhang et al., 2011), we identified the brain regions showing significant between-group differences in at least one nodal metric (*p* < 0.05, permutation corrected). We also performed the Pearson correlation analyses between the AUC of each network metric and the duration of *Baduk* training in BEs using SPSS (*p* < 0.05/110 for multiple comparisons correction).

## Results

### Gray matter volume

Relative to novices, BEs exhibited decreased GMV in the right AMY and increased GMV in the bilateral HOC, particularly the NA (Table 2; Figure 1A). Significant negative correlations were observed between the degree of GMV reduction in the mOFC adjacent to the gyrus rectus and training duration in BEs (*p* < 0.001, *r* = −0.802) (Table 2; Figures 1B,C).

### Functional connectivity

BEs showed increased FC in the right AMY seed and left mOFC, and decreased FC in the right NA seed and right mPFC compared to novices (Table 2; Figure 2B). We found no significant correlations between FC measures and training durations in BEs.

### Topological organization of the whole-brain network

#### Global network measures

Both BEs and novices showed small-world architecture in whole-brain functional networks (i.e., σ > 1). Compared to novices, BEs showed a lower normalized characteristic path length λ and increased normalized global efficiency *E*_glob_ in the whole-brain functional network (Table 4; Figure 3C). No significant differences were found in any other global network measures.

**Table 4:** **Regions showing significant differences in nodal centrality metrics between experts and novices**.

**Brain regions**	**Experts (*n* = 17)^†^**	**Novices (*n* = 16)^†^**	***p*-value^‡^**
**GLOBAL NETWORK METRICS**			
*Experts > Novices*			
Normalized global efficiency (E_glob_)	0.312 (0.003)	0.310 (0.003)	0.008
*Experts < Novices*			
Normalized characteristic path length (λ)	0.329 (0.003)	0.331 (0.004)	0.018
NODAL DEGREE^*^			
*Experts > Novices*			
Right postcentral gyrus	10.216 (2.768)	8.404 (2.969)	0.040
Right lateral occipital cortex, inferior division	9.637 (2.401)	7.288 (2.087)	0.002
Right thalamus	9.288 (2.842)	7.299 (2.812)	0.026
Left nucleus accumbens	8.458 (2.609)	6.117 (2.868)	0.011
Right nucleus accumbens	8.012 (2.469)	6.376 (2.653)	0.040
*Experts < Novices*			
Right superior frontal gyrus	7.892 (2.966)	10.223 (3.530)	0.025
Left inferior frontal gyrus, pars triangularis	7.983 (2.952)	9.826 (3.044)	0.044
Right inferior frontal gyrus, pars triangularis	8.839 (2.566)	10.640 (3.499)	0.050
Right middle temporal gyrus, posterior division	7.092 (1.931)	8.706 (2.313)	0.021
Left lateral occipital cortex, superior division	8.413 (2.554)	10.481 (2.537)	0.013
Right amygdala	7.002 (2.240)	8.391 (2.232)	0.043
NODAL EFFICIENCY^*^			
*Experts > Novices*			
Right postcentral gyrus	0.201 (0.016)	0.188 (0.019)	0.028
Right lateral occipital cortex, inferior division	0.198 (0.014)	0.184 (0.012)	0.002
Right cingulate gyrus, posterior division	0.194 (0.024)	0.178 (0.030)	0.038
Left thalamus	0.191 (0.019)	0.179 (0.018)	0.030
Right thalamus	0.195 (0.018)	0.182 (0.017)	0.024
Left nucleus accumbens	0.190 (0.016)	0.171 (0.028)	0.008
Right nucleus accumbens	0.188 (0.015)	0.174 (0.026)	0.029
*Experts < Novices*			
Right superior frontal gyrus	0.187 (0.019)	0.199 (0.022)	0.046
Right middle temporal gyrus, posterior division	0.184 (0.013)	0.192 (0.013)	0.048
Left lateral occipital cortex, superior division	0.191 (0.015)	0.201 (0.016)	0.043
NODAL BETWEENNESS^*^			
*Experts > Novices*			
Right postcentral gyrus	18.654 (9.765)	11.436 (7.248)	0.011
Right lateral occipital cortex, inferior division	17.396 (7.918)	11.238 (7.615)	0.015
Left intracalcarine cortex	12.856 (6.872)	8.879 (5.564)	0.044
Left parietal operculum cortex	22.417 (12.901)	15.055 (8.181)	0.025
Right thalamus	19.021 (14.113)	8.525 (4.291)	*p* < 0.001
*Experts < Novices*			
Right superior frontal gyrus	13.295 (7.365)	18.560 (9.814)	0.043
Right middle temporal gyrus, posterior division	12.337 (3.969)	15.264 (5.007)	0.035
Right middle temporal gyrus, temporooccipital part	10.113 (4.805)	15.355 (9.793)	0.030
Right supramarginal gyrus, posterior division	16.613 (7.972)	22.612 (9.574)	0.030
Left temporal fusiform cortex, anterior division	7.761 (6.532)	12.083 (7.511)	0.045
Left occipital fusiform gyrus	10.304 (5.000)	17.177 (13.525)	0.029
Right pallidum	8.257 (5.415)	14.800 (10.731)	0.014
Left amygdala	13.251 (8.945)	20.604 (13.487)	0.038

† *Values are presented as mean AUC (standard deviation) for each group*.

‡ *They are p-values based on nonparametric permutation tests*.

* *Regions were considered changed in experts if they exhibited significant between-group differences (p < 0.05, permutation-corrected) in at least one nodal metric*.

#### Regional network measures

**Figure 5:**
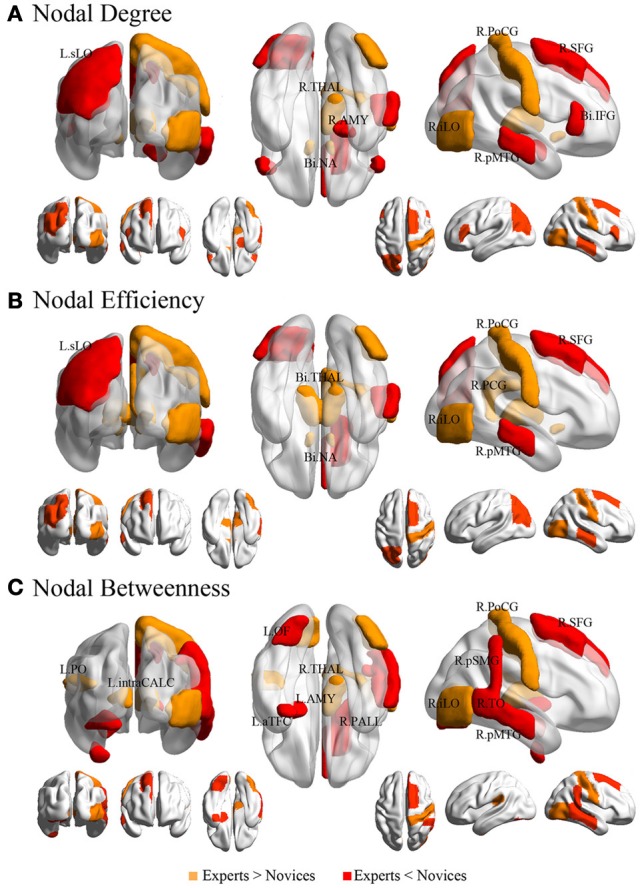
**Brain regions showing significant differences in each nodal metric between experts and novices.** Regions with significant group differences, including nodal degree (panel **A**), nodal efficiency (panel **B**), and nodal betweenness (panel **C**), were rendered using BrainNet Viewer (http://www.nitrc.org/projects/bnv/). See Table 4 for detailed information. R, right; L, left; Bi, bilateral; PoCG, postcentral gyrus; SFG, superior frontal gyurs; IFG, inferior frontal gyrus; pMTG, posterior division of middle temporal gyrus; sLO, superior division of lateral occipital cortex; iLO, interior division of lateral occipital cortex; THAL, thalamus; AMY, amygdala; NA, nucleus accumbens; PCG, posterior cingulate gyrus; pSMG, posterior division of supramarginal gyrus; OF, occipital fusiform gyrus; aTFC, anterior division of temporal fusiform cortex; PALL, pallidum; PO, parietal operculum cortex; TO, temporooccipital part of middle temporal gyrus; intraCALC, intracalcarine cortex.

The groups differed with respect to nodal centrality measures (i.e., nodal degree, nodal efficiency, and nodal betweenness) in several brain regions (Table 4; Figure 5). Compared to novices, nodal degree in BEs showed significant increases in the right postcentral gyrus (PocG), right inferior lateral occipital cortex (iLO), right thalamus, and bilateral NA, but significant decreases in the right superior frontal gyrus (SFG), bilateral inferior frontal gyrus (IFG), right posterior middle temporal gyrus (pMTG), left superior lateral occipital cortex (sLO), and right AMY (Figure 5A). In comparison to novices, nodal efficiency in BEs was significantly increased in the right PocG, right iLO, right posterior cingulate gyrus (PCG), bilateral thalamus, and bilateral NA, but significantly decreased in the right SFG, right pMTG, and left sLO (Figure 5B). Compared to novices, nodal betweenness in BEs was significantly higher for the right PocG, right iLO, left intracalcarine cortex, left parietal operculum cortex (PO), and right thalamus, while significantly lower for the right SFG, right pMTG, temporooccipital part of right middle temporal gyrus (TO), right posterior supramarginal gyrus (pSMG), left anterior temporal fusiform cortex (aTFC), left occipital fusiform gyrus (OF), right pallidum, and left AMY (Figure 5C).

**Figure 6:**
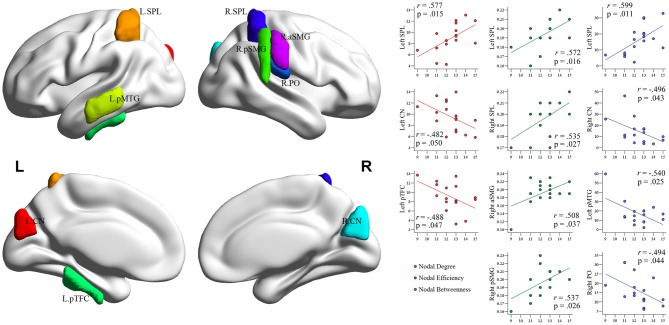
**Plots of the correlations between each nodal metric and training duration of experts.** The value of regional nodal measures (red, nodal degree; green, nodal efficiency; blue, nodal betweenness) for each BEs is shown on the *x*-axes and duration of *Baduk* training (in years) on the *y*-axes. All these correlations did not survive multiple comparisons correction.

Nodal network metrics in several regions were correlated with training duration of BEs (*p* < 0.05; Figure 6), although there were no correlations between brain regions showing significant group difference in nodal network metrics and training durations of BEs. The duration of training in BEs was positively correlated with nodal degree in the left SPL, but negatively correlated with nodal degree in the left CN and left pTFC. Nodal efficiency in the bilateral SPL, right anterior supramarginal gyrus (aSMG), and right pSMG was positively correlated with training duration. Training duration in BEs was positively correlated with nodal betweenness in the left SPL, but negatively correlated with nodal betweenness in the right CN, left pMTG, and right PO. Figure 6 shows plots of the correlation between nodal metrics and training duration in BEs. However, all of these correlations did not withstand correction for multiple comparisons (*p* < 0.05/110).

## Discussion

To our knowledge, this is the first study to combine structural MRI and rs-fMRI to investigate the morphological differences in the brain of BEs, the effect of such morphological differences to functional circuits at rest, and the topological organization of the whole-brain functional network in board-game experts, who are treated as excellent examples of cognitive expertise. Four main findings emerged from this study. First, relative to novices, BEs showed increased GMV in the bilateral NA and reduced GMV in the right AMY. Additionally, the GMV in the mOFC was correlated with training duration. Second, BEs showed higher FC between the right AMY and left mOFC, and decreased FC between the right NA and right mPFC. Third, BEs showed increased global efficiency and decreased characteristic path length, implying an increase in the global integration of the whole-brain functional network. Fourth, BEs exhibited differences in the nodal centrality metrics of many brain regions related to the diverse cognitive functions utilized in *Baduk* games.

### Intuitive expertise in board-game experts and its implication on the present findings

Board games have historically been the primary focus of research on expertise (de Groot, 1965; Chase and Simon, 1973; Kahneman and Klein, 2009). Specifically, interest in how board-game experts make rapid and effective decisions and in the neural correlates associated with these intuitive judgments has been increasing in recent years (Duan et al., 2012; Wan et al., 2012). Such expertise or unconscious processing and linked brain changes are the products of prolonged experience within a domain and cannot be obtained through shortcuts. As it was previously stated, researchers have studied intuitive expertise in board-game experts using the NDM approach, particularly the RPD model (de Groot, 1965; Klein, 1998, 2008; Kahneman and Klein, 2009). To describe how experts can make extremely rapid and good decisions, the RPD model combines intuitive pattern matching processes based on past experience and deliberative mental simulation processes (Klein, 2008; Kahneman and Klein, 2009). These two processes correspond to System 1 and System 2, respectively, in dual-process accounts of cognition (Hodgkinson et al., 2009; Kahneman and Klein, 2009). Interestingly, in this study, brain regions showing significant group difference in GMV, the NA and AMY, correspond to theories of the psychological and neurobiological mechanisms underlying intuitive judgment. That is, these regions belong to the X-system (System 1), which supports reflexive cognitive processing in social cognition (Lieberman, 2007) and these regions, as expected, also belong to the somatic marker circuitry based on the SMH. Compared with novices, BEs showed reduced GMV in the AMY and increased GMV in the NA. This finding is very interesting because of the inverse effect (increase/decrease) on the GMV of these two regions. Previous studies have reported specific correlations between regional cortical thickness and each component of cognition (Westlye et al., 2011), as well as a mixed pattern of increases and decreases in regional cortical thickness in experts (Kang et al., 2013). For example, meditation experts showed a thicker cortex in the mOFC and temporal pole, areas associated with emotional processing, and a thinner cortex in the parietal areas and PCG, areas associated with attention and self-perception when compared with novices (Kang et al., 2013). In addition, recent studies have argued for the nonlinearity of training-induced GMV changes, showing an initial increase followed by a decrease in regional GMV (Boyke et al., 2008; Driemeyer et al., 2008), and suggested that these changes are affected by training length and intensity (Takeuchi et al., 2011). Based on such previous studies, a possible explanation for the inverse effect of the AMY and NA on the GMV is that each component involved in *Baduk* expertise affects a different part of the brain in a distinctive way (e.g., increase/decrease on GMV), or that specific processing related to how the amygdala shows reduced GMV may be more strongly involved in BEs.

Contrary to our expectations, BEs and novices did not show any differences in the GMV of the occipitotemporal and parietal areas associated with visuospatial processing and spatial attention. However, we found significant group differences in terms of regional nodal properties of the functional brain network in these regions, particularly in the iLO, pSMG, TO, and pMTG, as well as in the NA and AMY, showing morphological differences. Previous studies have reported that compared with novices, long-term chess experts (over 10 years of training) have morphological and functional differences in the HOA, showing decreased GMV and increased FC in brain regions known as the default-mode network (DMN) during resting-state in the region (Duan et al., 2012), whereas individuals who trained for 15 weeks showed difference only in the activity of anatomically identified HOA but not in morphological differences in the region between before and after training, exhibiting increased activity during chess play (Wan et al., 2012). Based on previous studies and our present findings, it is, thus, conceivable that changes in brain structure, as well as those in brain function, through long-term training may be required to become experts (i.e., to reach a professional level), and that BEs may primarily be involved in intuitive decision-making, which is associated with the regions showing both morphological and FC differences, and secondarily in visuospatial processing, which is associated with the regions showing group differences only in the functional brain network.

### Structural and functional differences in the amygdala

The observed decrease in GMV of the AMY following training is consistent with results from previous studies that involved cognitive, motor, or mental training (Boyke et al., 2008; Takeuchi et al., 2011; Kang et al., 2013), suggesting that a possible mechanism underlying such structural changes is the use-dependent selective elimination of synapses (Huttenlocher and Dabholkar, 1997). The AMY is involved in configural/holistic visual processing for both faces and emotional facial expressions (Sato et al., 2011); it enables people to master facial affective processing. More specifically, the right AMY is more relevant to the unconscious (Morris et al., 1998) and rapid (Wright et al., 2001) processing of facial expressions than the left AMY. Beyond its role in emotional and facial processing, the AMY, in addition to the VMPFC, has also been proposed as one of key structures in the SMH and decision-making, as measured by the Iowa gambling task (Bechara et al., 2005), a decision-making task that requires implicit learning and executive function abilities. Additionally, animal studies have found that the AMY may participate in the recognition of visual patterns based on past experiences through direct thalamo–amygdala projection (LeDoux et al., 1989), and that lesions to this area may increase impulsivity in decision-making (Winstanley et al., 2004). Human patients with AMY damage have shown decreased decision-making performance under conditions of ambiguity and risk (Brand et al., 2007). Thus, GMV reduction in the AMY of BEs may be related to intuitive decision-making that is based on feelings (a somatic marker) rather than on reasoning and/or the enhanced cognitive functioning (e.g., holistic visual processing) achieved through long-term *Baduk* training. The results of our RSFC and correlation analyses support this interpretation by showing increased FC between the AMY and mOFC in BEs and a correlation between the GMV in the mOFC and training duration. Previous studies have demonstrated that interactions between the AMY and mOFC are crucial for goal-directed behavior (Holland and Gallagher, 2004) and emotional regulation (Banks et al., 2007). Lee et al. (2010) recently reported differences in the WM tract, the uncinate fasciculus, connecting these two regions in BEs. As mentioned above, the mOFC/vmPFC is an important area that generates somatic markers based on the SMH, and correlation between the GMV in the region and training duration suggests that morphological difference in the region may be contributed by *Baduk* training rather than pre-existing differences before training. Contrary to this interpretation, an alternative one of the present finding is that this opposing pattern (reduced GMV and increased FC in the amygdala) may reflect a compensatory neural mechanism pre-existing in people who later become BEs. That is, the observed pattern may be an endophenotype that enables improved somatic-marker-based (automatic) decision-making, and thus favorably influences (i.e., predicts) the development of *Baduk* expertise.

### Structural and functional differences in the striatum

In contrast to the GMV in the AMY, the GMV in the NA was increased in BEs compared to novices. This is especially intriguing, as a recent study with chess experts using the same method reported a GMV decrease in the dorsal part of HOC, which is known to be involved in the quick generation of the best next move during chess playing (Wan et al., 2011), while we found a GMV increase in the ventral part of the HOC, the NA (Duan et al., 2012). Whereas the dorsal striatum is associated with sensorimotor experiences or reward-outcomes processing, the ventral striatum is associated with emotional and motivational experiences or reward-anticipation processing. Consistent with the present finding, Boyke et al. (2008) found increased GMV in the NA after juggling training. They suggested that learning to juggle may stimulate an increase in the size of this region due to its role as an interface between the limbic and motor systems rather than any role in motor control (Mogenson et al., 1980).

We also found decreased FC between the NA and mPFC in BEs compared with novices. Such connections are seen in the anterior part of the DMN (Di Martino et al., 2008; Duan et al., 2012; Jung et al., 2013), which is thought to be involved in self-referential processing and theory of mind (ToM) (Murdaugh et al., 2012; Reniers et al., 2012). ToM refers to the ability to infer the thoughts or intentions of others. Given the hyperconnectivity within the DMN of individuals at high-risk for psychosis (Shim et al., 2010), who also suffer from impaired ToM (Chung et al., 2008), a reverse pattern (decreased FC) in BEs may be due to their capacity to infer the opponent's intention while playing *Baduk*. It is also conceivable that the strength of this connection may reflect a change in their sensitivity to feedback, given that these couplings are thought to alter expectations in the face of negative feedback (van den Bos et al., 2012). However, these ideas are just speculations, and additional neuroimaging studies with *Baduk* tasks are necessary to identify the physiological mechanisms that underlie these brain differences and to clarify the relationship between their cognitive functions and brain structure and function.

### Topological alteration in the whole-brain functional network

The GTA results reveal increased global efficiency and decreased characteristic path length in Bes compared with novices, implying an increase in the global integration of the whole-brain network. This can be reflective of effective integrity and rapid information propagation between and among the remote regions of the brain involved in the cognitive processing required for *Baduk* play in BEs (Wang et al., 2010). Thus, this finding reflects a difference in the functional aspect of the whole-brain circuitry in the service of achieving the most efficient network for playing *Baduk*.

We also found the following differences between the two groups in the regional nodal characteristics of many brain regions: increased nodal centrality metrics in nine regions, namely the right PocG, right iLO, right PCG, left intracalcarine cortex, left PO, bilateral NA, and bilateral thalamus, and decreased nodal centrality metrics in 12 regions, namely the right SFG, right pMTG, right TO of MTG, right pSMG, right pallidum, left sLO, left aTFC, left OF, bilateral IFG, and bilateral AMY. Interestingly, whereas the brain regions showing increased nodal centralities in BEs were involved primarily in implicit processing, such as somatic sensation (PocG and thalamus), visual expertise (iLO), and affective/motivational processing (NA), the brain regions showing decreased nodal centralities were related to higher-order cognitive functions such as executive function (SFG, IFG, and sLO), semantic memory processing (pMTG and pSMG), and visual perception (TO and OF). It is speculated that brain regions showing differences in nodal centralities are important contributors in *Baduk* expertise, and may facilitate efficient exchange of information. In this context, our findings are consistent with the RPD model mentioned above (Klein, 2008) Therefore, *Baduk* is thought to involve diverse cognitive functions with respect to both automatic and deliberative processes.

Previous neuroimaging studies that focused on *Baduk* reported enhanced activation in the occipitotemporal and parietal cortices, during these games (Chen et al., 2003; Ouchi et al., 2005), as well as learning-induced differences in activity in fronto–parietal and visual cortices (Itoh et al., 2008), which corresponds to our findings of differences in the PocG and iLO. Correlation analyses between each nodal metric and training duration of BEs showed positive correlations between these two values in the parietal areas, particularly more extensive in the right than left hemisphere, although it did not remain significant after correction for multiple comparison. The right SPL in terms of *Baduk* play, may contribute to spatial working memory (Ungerleider et al., 1998) and/or spatial attention (Fink et al., 1996).

Chess requires the recognition of the identity and function of each piece (i.e., chess-specific object and function recognition, which is known to be associated with the occipito-parieto-temporal junction, OTJ; Bilalić et al., 2011a,b), whereas this is not needed in *Baduk*. This difference as a confounding factor makes it difficult to compare or interpret the results of the present study with those of previous neuroimaging studies on chess experts. However, both *Baduk* and chess involve pattern recognition with respect to the spatial positioning of stones or pieces, which is an essential component in improving both games. Recently, Bilalić et al. (2010, 2011b) demonstrated that the CoS and RSC, part of the parahippocampal place area, play an important role in chess-specific pattern recognition. Intriguingly, we found increased nodal centralities in the iLO, which corresponds to the OTJ associated with chess-specific object and function recognition in studies by Bilalić et al. (2011a,b), in BEs compared to novices but not any differences in the Cos and RSC between the two groups. This discrepancy in results between the present study with BEs and previous studies with chess experts may stem from differences between the basic aspect of pattern recognition in these board games; pattern recognition in *Baduk* is only based on the shapes the stones take, while that in chess is based on object and function recognition of chess pieces and their ability to rapidly access the information of potential moves or move sequences for each piece. Thus, differences in the iLO may be linked to the recognition of shapes and integration of local features (Kourtzi et al., 2003) during *Baduk* play.

Taken together, these GTA results provide such insight into the topological organization of the functional brain networks of BEs: increased functional integration across global brain regions and increased nodal centralities in regions associated with spatial attention and somatic sensation.

### Limitations

The present study has some limitations. First, it is difficult to accurately and quantitatively assess the skill level of BEs. Although we used training duration (in years) as a proxy for skill level in BEs, the validity of this proxy can be challenged. Skill levels in BEs may be independent of training duration, resulting in similar skill levels for any given pair of short- and long-trained participants. Previous studies have described that the lack of any significant correlation between brain imaging data and the amount/intensity of training in experts may result from the inaccuracy of the proxy chosen in determining the actual extent/intensity of the individual training (Luders et al., 2011; Kang et al., 2013). Second, as a result of the cross-sectional nature of this study, our findings do not allow any unambiguous definitive causality. Therefore, it is unclear whether the brain differences we found result from acquiring *Baduk* expertise through prolonged training, or if they simply reflect pre-existing differences in brain structure and function that predict later expertise. Longitudinal studies will help to clarify this issue. Finally, the present findings with respect to FC and GTA are based on rs-fMRI data of BEs rather than on brain activity during game performance. Thus, further functional imaging studies are necessary to investigate the topological properties and FC within the functional brain network while the individuals actually play *Baduk*.

## Conclusions

The current study demonstrates differences in the structure and the functional circuits of the AMY and NA in BEs; compared with novices, experts showed decreased GMV and increased FC with the mOFC in the AMY as well as increased GMV and decreased FC with the mPFC in the NA. As interfaces between the cognitive and affective components of the limbic cortico–striatal loop, the AMY and NA are involved in implicit processing and goal-directed adaptive behavior under changing environmental conditions. In particular, the AMY is critical for emotional and holistic visual processing, as well as emotion-based decision-making. Based on our hypothesis that long-term *Baduk* training would influence the structure and functional circuits of regions associated with the cognitive mechanisms underlying *Baduk* expertise, our findings suggest that intuitive decision-making, which is mediated by somatic marker circuitry such as the AMY and NA, is a key cognitive component of *Baduk* play. The current study also provides new evidence for differences in the topological organization of the whole-brain network of BEs, showing increased global integration and altered regional nodal centralities in the regions related to visuospatial processing.

### Conflict of interest statement

The authors declare that the research was conducted in the absence of any commercial or financial relationships that could be construed as a potential conflict of interest.
